# Tolerogenic dendritic cells are efficiently generated using minocycline and dexamethasone

**DOI:** 10.1038/s41598-017-15569-1

**Published:** 2017-11-08

**Authors:** Jae-Hee Lee, Chan-Su Park, Sundong Jang, Ji-Wan Kim, Sang-Hyeon Kim, Sukgil Song, Kyungjae Kim, Chong-Kil Lee

**Affiliations:** 1 College of Pharmacy, Chungbuk National University, Cheongju, 28644 South Korea; 2 College of Pharmacy, Sahmyook University, Seoul, 01795 South Korea

## Abstract

Tolerogenic dendritic cells (tDCs) represent a promising tool for cellular therapy against autoimmune diseases, allergies, and transplantation rejection. Numerous pharmacological agents are known to induce tDC generation. Minocycline, which has long been used as a broad-spectrum antibiotic, was recently shown to significantly increase the generation of DCs with regulatory properties. Here, we examined the effect of the combination of minocycline with dexamethasone, rapamycin, vitamin D3, and interleukin (IL)-10, which are all known inducers of tDC generation. The highest number of tDCs was generated when minocycline and dexamethasone were used together with granulocyte colony-stimulating factor (GM-SCF) and IL-4. The tolerogenicity of the minocycline/dexamethasone-conditioned tDCs was much better than or at least equal to those of the tDCs generated with either one of these agents, as assessed through *in vitro* phenotypic and functional assays. In addition, pretreatment with MOG35-55 peptide-pulsed minocycline/dexamethasone-conditioned tDCs significantly ameliorated the clinical signs of experimental autoimmune encephalitis induced by MOG peptide injection in a murine model. These results confirmed that tDCs with potent tolerogenic properties could be efficiently generated by the combined use of minocycline and dexamethasone, along with GM-CSF and IL-4. Our results would help in the development of *ex vivo* tDC-based immunotherapies.

## Introduction

Dendritic cells (DCs) are highly specialised antigen presenting cells (APCs), which are crucial for the induction of immune responses and the development and maintenance of immune tolerance^1–3^. The functional difference in DCs, immunogenic versus tolerogenic, depends on the maturation state and maturation environment. In the presence of activation-inducing stimuli such as inflammatory cytokines, CD40 ligands, or lipopolysaccharide (LPS), immature DCs become potent stimulators of T cell-mediated responses by upregulating MHC class-II and co-stimulatory molecules^4,5^.

Tolerogenic DCs (tDCs) are induced by immunosuppressive cytokines such as interleukin (IL)-10 and transforming growth factor (TGF)-β, as well as by various pharmacological agents, including dexamethasone, rapamycin, vitamin D3, aspirin, atorvastatin, retinoic acid, and mycophenolic acid, when used together with granulocyte colony-stimulating factor (GM-CSF) and IL-4^6^. tDCs are characterised by reduced expression of costimulatory molecules and IL-12, decreased ability to induce T-cell proliferation, increased IL-10 secretion, and increased induction of regulatory T cells (Tregs)^7–9^. The mechanisms underlying tDC activity include the induction of Tregs, increasing the expression of programmed death-ligand 1 (PD-L1) and inducible T-cell co-stimulator ligand (ICOSL), and the production of immunosuppressive factors such as IL-10 and TGF-β^7–11^.

Antigen (Ag)-specific tolerance has been shown to be induced *in vivo* through vaccination with Ag-pulsed *ex vivo*-generated tDCs in experimental animals as well as in humans^12–16^. One of the major concerns in tDC-based immunotherapy is the generation of sufficient tDCs with stable tolerogenic properties at a reasonable cost. For instance, rapamycin (10 ng/ml) is effective in generating tDCs from bone marrow cells when used together with GM-CSF and IL-4. However, the number of CD11c^+^ cells obtained from rapamycin-conditioned cultures is significantly (more than 40%) lower than that from rapamycin-unconditioned cultures^7^. Dexamethasone has also been shown to markedly reduce DC recovery^17,18^. Minocycline, a second-generation semi-synthetic tetracycline that has been clinically used for over 30 years as an antibiotic, has been shown to promote the generation of DCs with tolerogenic properties when used together with GM-CSF and IL-4^19^.

In the present study, we examined the effect of the combination of minocycline with dexamethasone, rapamycin, vitamin D3, and IL-10, all of which are known to induce tDCs in mouse and human systems. Our results demonstrated that the combination of minocycline and dexamethasone was optimal for the generation of a large number of tDCs with potent tolerogenicity. Both minocycline and dexamethasone are inexpensive and have been proven to be safe in the clinic. Our study would be of great help for the development of *ex vivo*-generated tDC-based immunotherapies.

## Results

### Combination of minocycline and dexamethasone generates the largest number of tDCs

**Figure 1:**
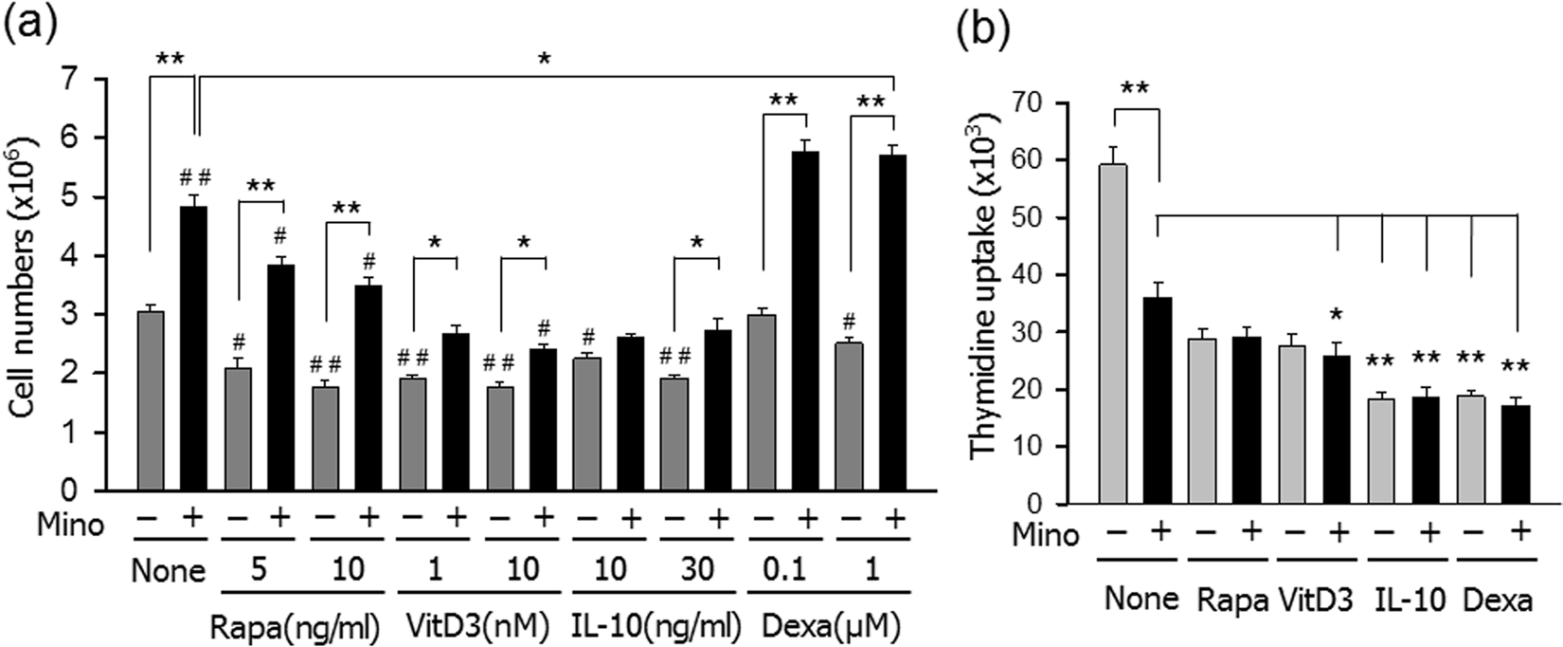
Effects of minocycline alone and in combination with rapamycin, vitamin D3, IL-10, or dexamethasone on the generation and allostimulatory capacity of DCs. (**a**) C57BL/6 bone marrow cells were cultured in a culture medium with 40 ng/ml GM-CSF and 40 ng/ml IL-4. Minocycline (5 μM) was added at the start of the culture, and the indicated amounts of rapamycin, vitamin D3, or IL-10 were added from day 3. On day 7, cells were harvested and counted using a haemocytometer. The data are presented as the mean ± standard deviation (SD) of six independent experiments. One way ANOVA tests were performed in order to evaluate significance. ^#^P < 0.05, ^##^P < 0.01 compared with untreated control. *P < 0.05, **P < 0.01 compared with matched group. (**b**) DCs generated from C57BL/6 mouse bone marrow cells were exposed to 50 ng/mL IFN-γ plus 50 ng/mL TNF-α for 24 h to induce maturation, and then co-cultured for three days with CD4^+^ T cells isolated from the spleens of BALB/c mice at 1:10 ratio. The cell proliferation was measured by the incorporation [^3^H]-thymidine added before the final 18 h of culture. The data are presented as the mean ± SD of three independent experiments. *P < 0.05, **P < 0.01 compared with matched group in one way ANOVA.

Bone marrow cell-derived DCs (control DC, cDC) were generated by culturing with GM-CSF and IL-4, and tDCs were generated by adding minocycline alone and in combination with rapamycin, vitamin D3, IL-10, and dexamethasone. Minocycline was added from the initiation of the culture, while the other agents were added on day 3 of the culture. On day 7, the DCs were harvested and counted. In the absence of minocycline, the number of generated DCs significantly decreased after the addition of rapamycin, vitamin D3, IL-10, or dexamethasone, compared to the control DCs (Fig. 1a). The addition of minocycline increased the number of generated DCs not only in the controls, but also in the cultures treated with rapamycin, vitamin D3, IL-10, or dexamethasone (Fig. 1a). The most significant increase was observed when minocycline was used in combination with dexamethasone. The addition of minocycline to the dexamethasone-treated culture increased the generation of DCs to about 220% (P < 0.01), compared to the dexamethasone-only culture. The DC proliferation-inducing activity of minocycline was also confirmed by both XTT assay and ^3^H-thymidine uptake assay (Supplement 1).

The allostimulatory capacity of the cDCs and the tDCs were also compared (Fig. 1b). For this comparison, tDCs generated with 10 ng/ml rapamycin, 10 nM vitamin D3, 30 ng/ml IL-10, or 1 μM dexamethasone in the presence or absence of 5 μM minocycline were used. Compared to the control DCs, DCs generated with minocycline alone were significantly deficient in allogeneic T cell stimulation. DCs generated with minocycline and IL-10, as well as with minocycline and dexamethasone, were even more deficient in allogeneic T cell stimulation than DCs generated with minocycline alone. Based on these results and the fact that dexamethasone is much more chief than IL-10 to enhance DC proliferation, we decided to generate tDCs with minocycline (5 μM), dexamethasone (1 μM), or both, and compared their tolerogenicity, both *in vitro* and *in vivo*.

### Minocycline/dexamethasone-conditioned tDCs are severely deficient in Ag-specific T-cell stimulation and inflammatory cytokine production, but produce high amounts of IL-10

**Figure 2:**
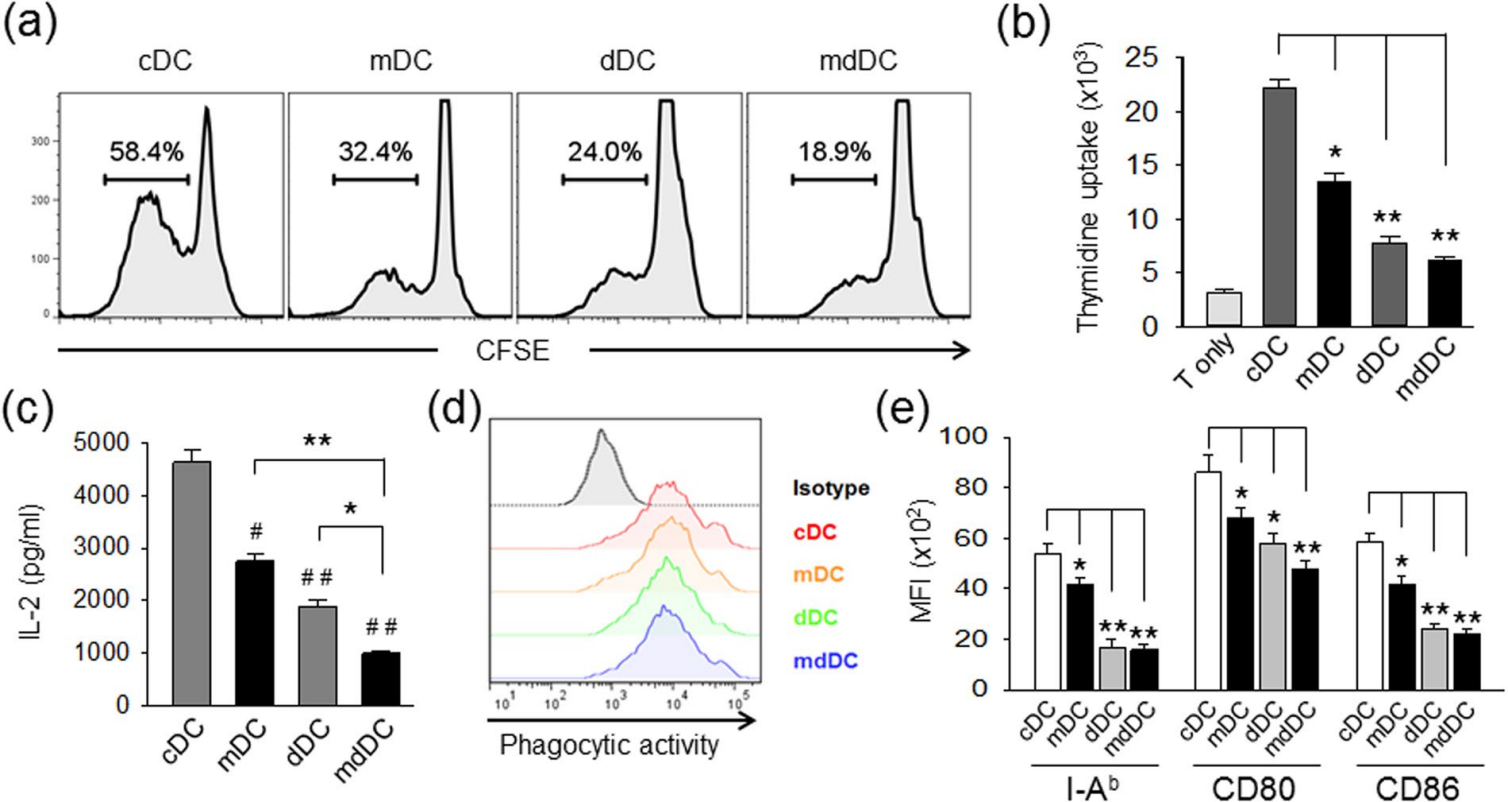
Suppressed antigen-presenting cell functions of minocycline/dexamethasone-conditioned tDCs. (**a**) DCs were generated in the absence of additional stimulus (cDC), or in presence of minocycline (mDC), dexamethasone (dDC), or both (mdDC), by culturing for 7 days. DCs were harvested, matured, pulsed with OVA_323–339_ peptide, and then co-cultured with CFSE-labelled CD4^+^ T cells isolated from OT-II mice for 3 days. Cell proliferation was analysed by flow cytometry. (**b**) Cell proliferation in experimental setting of (**a**) was measured by incorporating [^3^H]-thymidine added before the final 18 h of culture. (**c**) Immature DCs were incubated with OVA-microspheres (50 μg/ml as OVA) for 1 h. After washing and fixing, cells were co-cultured with OVA_323–339_ peptide-specific DOBW cells. The amount of IL-2 in the culture supernatants were measured by ELISA. The data are presented as the mean ± SD of five independent experiments. (**d**) Phagocytic activity of DCs harvested on day 7 was measured by flow cytometry after incubating the DCs with FITC-labelled OVA-microspheres for 1 h. (**e**) Mature DCs were analysed for the expression I-A^d^, CD80, and CD86 by flow cytometry. One way ANOVA tests were performed in order to evaluate significance. ^#^P < 0.05, ^##^P < 0.01 compared with cDC group. *P < 0.05, **P < 0.01 compared with the matched group.

The ability of DCs generated in the presence of minocycline (mDC), dexamethasone (dDC), and both (mdDC) to stimulate Ag-specific T-cell proliferation was compared by co-culturing OVA_323-339_ peptide-pulsed DCs with CFSE-labelled CD4^+^ T cells isolated from OT-II mice. The proportion of proliferated CD4^+^ T cells was significantly lower in the mdDC co-culture (18.9%) than in the cDC co-culture (58.41%), mDC co-culture (32.4%), or dDC co-culture (24.0%) (Fig. 2a). The proliferation of OVA_323-339_ peptide-specific CD4^+^ T cells was quantitatively compared based on the [^3^H]-thymidine uptake, and it was confirmed that mdDCs were the most deficient in inducing Ag-specific T-cell proliferation (Fig. 2b).

The APC function of DCs was determined by inducing the phagocytosis of ovalbumin (OVA)-microspheres. After a 2-h incubation with OVA-microspheres, the DCs were washed, fixed and analysed for OVA-specific CD4 T cell stimulatory capacity by measuring the IL-2 production by OVA-specific CD4^+^ T cell hybridoma DOBW cells, which recognise OVA[323-339]-I-A^d^ complexes and secrete IL-2, after co-incubation with DOBW cells. mdDCs were found to be the least efficient in inducing IL-2 production from DOBW cells (Fig. 2c). The differences in MHC class-II-restricted antigen presentation, however, could not be attributed to the phagocytic activity, which was found to be very similar in all the populations (Fig. 2d).

**Figure 3:**
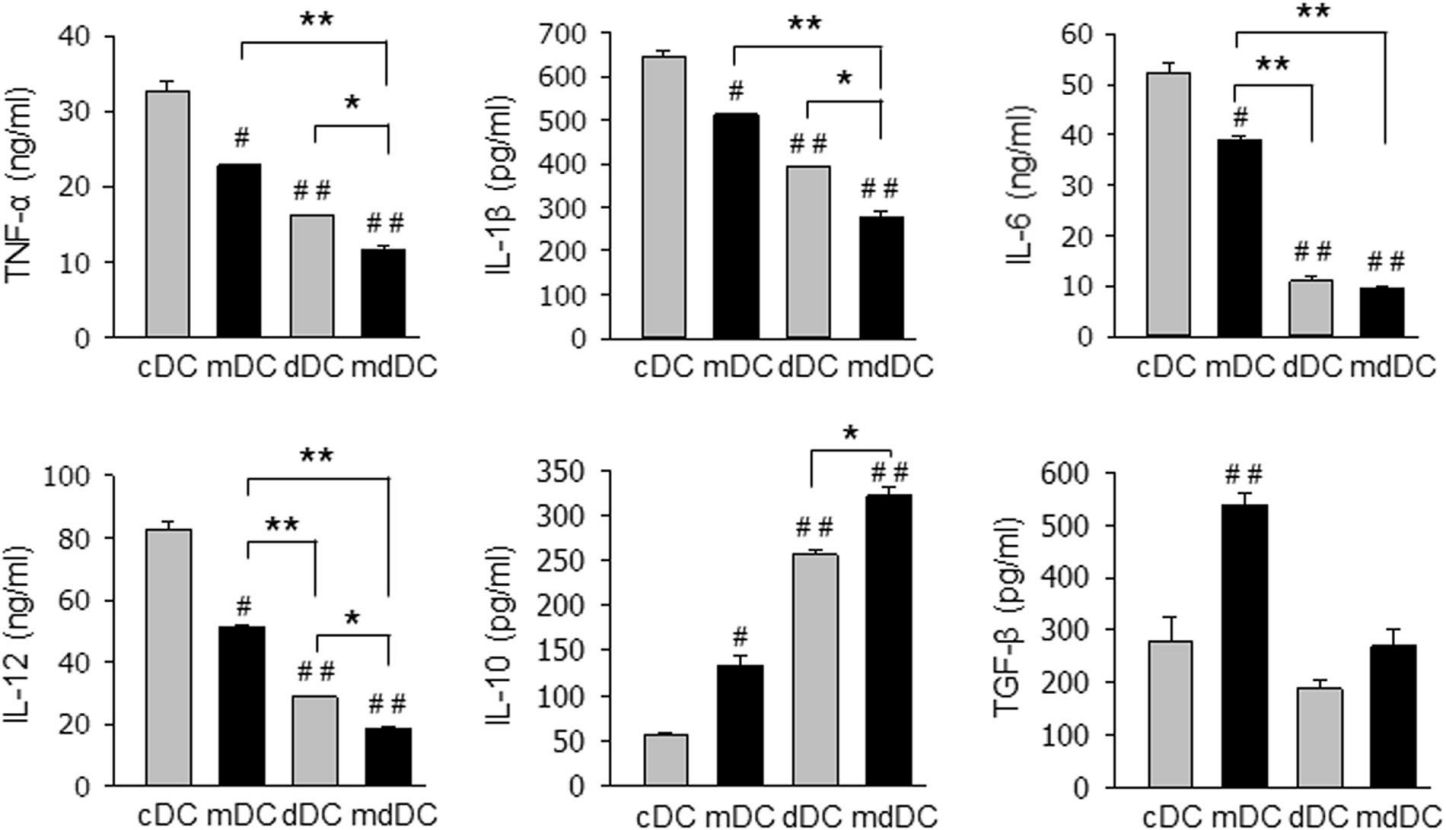
Suppressed cytokine production of minocycline/dexamethasone-conditioned tDCs. DCs (2 × 10^5^ cells/well) were stimulated with LPS for TNF-α production, or IFN-γ plus TNF-α for the other cytokine production, for 24 h, and cytokine secretion in the supernatant was determined by ELISA. The data are presented as the mean ± SD of at least three independent experiments. One way ANOVA tests were performed in order to evaluate significance. ^#^P < 0.05, ^##^P < 0.01 compared with cDC group. *P < 0.05, **P < 0.01 compared with matched group.

The expression levels of the co-stimulatory molecules of the DCs were compared after maturation with IFN-γ and TNF-α. mdDCs expressed the lowest levels of MHC class II (I-A^b^), CD80, and CD86 among all the DC populations (Fig. 2e). In addition, mdDCs were resistant to maturation induced by IFN-γ and TNF-α (Supplemetary Figure S2). Furthermore, mdDCs produced significantly lower levels of proinflammatory cytokines, TNF-α, IL-1β, and IL-12, than mDCs or dDCs (Fig. 3). The IL-6-producing capability of mdDCs was also significantly lower than that of mDCs. There was no difference in IL-6-producing capability between dDCs and mdDCs, probably due to profound suppression in both DCs. In contrast, the IL-10-producing capability of mdDCs was significantly higher than that of mDCs, and dDCs. Surprisingly, the TGF-β-producing capability was significantly higher in mDCs than that of cDCs, and was suppressed by culturing the DCs in the presence of dexamethasone. mdDCs produced almost the same levels of TGF-β compared to cDCs (Fig. 3).

### Minocycline/dexamethasone-conditioned tDCs induce Foxp3^+^ Tregs

**Figure 4:**
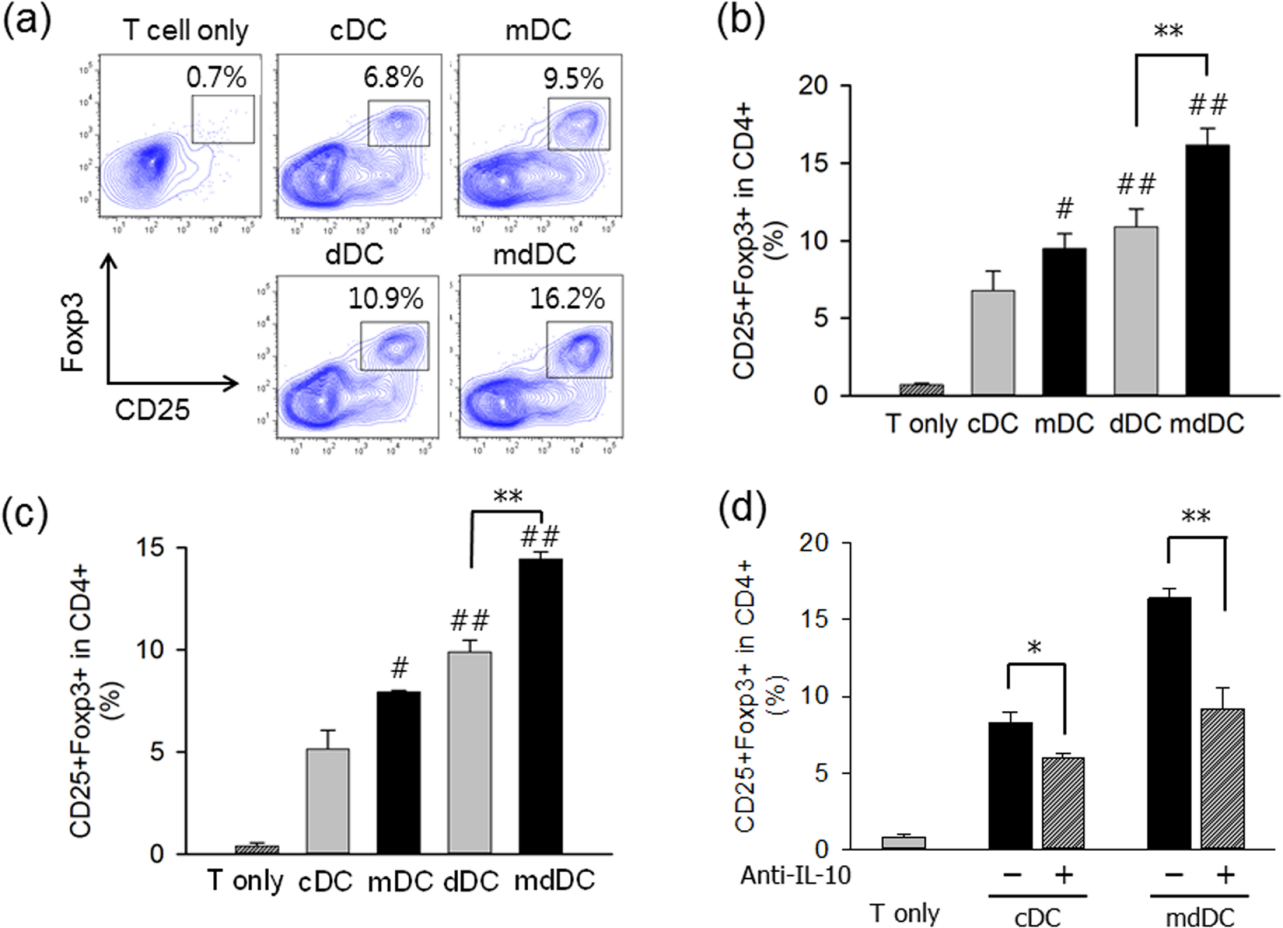
Induction of Foxp3^+^ Tregs by tDCs. (**a**) Generation of Foxp3^+^ Tregs from CD4^+^CD25^−^ OT-II T cells. Mature cDCs, mDCs, dDCs, or mdDCs generated from BM cells of C57BL/6 mice were pulsed with the OVA_323–339_ peptide (10 μg/ml), washed, and co-cultured with CD4^+^CD25^−^ T cells isolated from OT-II mice in the presence of 100 U/ml IL-2. After 4 days, cells were analysed for the expression of CD25 and Foxp3. Representative histograms are shown. (**b**) The proportion of CD25^+^Foxp3^+^ T cells in the CD4^+^ cell population of each experimental group of (a) is shown. The data present the mean ± SD of three independent experiments. (**c**) Generation of Foxp3^+^ Tregs in allogenic MLR. Mature cDCs, mDCs, dDCs, or mdDCs generated from BM cells of C57BL/6 mice were cultured with naïve CD4^+^CD25^−^ T cells isolated from the spleens of BALB/c mice for 4 days in a medium containing 10 U/ml of recombinant human IL-2. After 4 days, cells were analysed for the expression of CD25 and Foxp3. The proportion of CD25^+^Foxp3^+^ T cells in the CD4^+^ cell population of each experimental group is shown. The data present the mean ± SD of three independent experiments. (**d**) Effects of anti-IL-10 neutralizing monoclonal antibody on the generation of Foxp3^+^ Tregs from CD4^+^CD25^−^ OT-II T cells. In the experimental system of (a), anti-IL-10 monoclonal antibody, or isotype-matched monoclonal antibody (10 μg/ml) was added from the initiation of the culture. The data present the mean ± SD of three independent experiments. One way ANOVA tests were performed in order to evaluate significance. ^#^P < 0.05, ^##^P < 0.01 compared with cDC group. *P < 0.05, **P < 0.01 compared with matched group.

The ability of DCs to induce the conversion of naïve CD4^+^ CD25^−^ T cells into Foxp3^+^ Tregs was compared *in vitro*. DCs derived from C57BL/6 mice (H-2^b^) were matured with IFN-γ and TNF-α, pulsed with OVA323–339 peptides, washed, and then co-cultured with CD4^+^CD25^−^ T cells isolated from OT-II mice in the presence of 100 U/ml of IL-2. After 4 days, the cells were harvested and stained with monoclonal antibodies specific for mouse CD4, CD25, and Foxp3. The cells were then gated on CD4^+^ cells and analysed for the expression of CD25 and Foxp3 (Fig. 4a). The proportion of CD25^+^Foxp3^+^ T cells in the CD4^+^ T cell population was significantly higher in mDC (9.5%, P < 0.05), dDC (10.9%, P < 0.01), and mdDC co-cultures (16.2%, P < 0.01), than in the cDC co-cultures (6.8%) (Fig. 4b).

The ability of DCs to induce the conversion of naïve CD4^+^ CD25^−^ T cells into Foxp3^+^ Tregs was also examined in allogeneic MLR. In this experiment, DCs derived from C57BL/6 mice (H-2^b^) were matured with IFN-γ and TNF-α, and then co-cultured with CD4^+^CD25^−^ T cells isolated from BALB/c mice (H-2^d^) in the presence of 10 U/ml of IL-2. After 4 days, the cells were harvested and stained for the expression of CD4, CD25, and Foxp3. The proportion of CD25^+^Foxp3^+^ T cells in the CD4^+^ T cell population was significantly higher in mDC (8.0%, P < 0.05), dDC (9.9%, P < 0.01), and mdDC co-cultures (14.5%, P < 0.01), than in the cDC co-cultures (5.2%) (Fig. 4c).

To investigate the mechanism underlying increased production of Tregs by mdDCs, we performed IL-10 blocking experiment using IL-10 neutralizing monoclonal antibody. DCs derived from C57BL/6 mice (H-2^b^) were matured with IFN-γ and TNF-α, pulsed with OVA323–339 peptides, and then co-cultured with CD4^+^CD25^−^ T cells isolated from OT-II mice in the presence of anti-IL-10 monoclonal antibody or isotype-matched control antibody (10 μg/ml) for 4 days. Addition of anti-IL-10 reduced the mdDC-induced generation of CD25^+^Foxp3^+^ T cells to approximately 60% (P < 0.01) (Fig. 4d)

**Figure 5:**
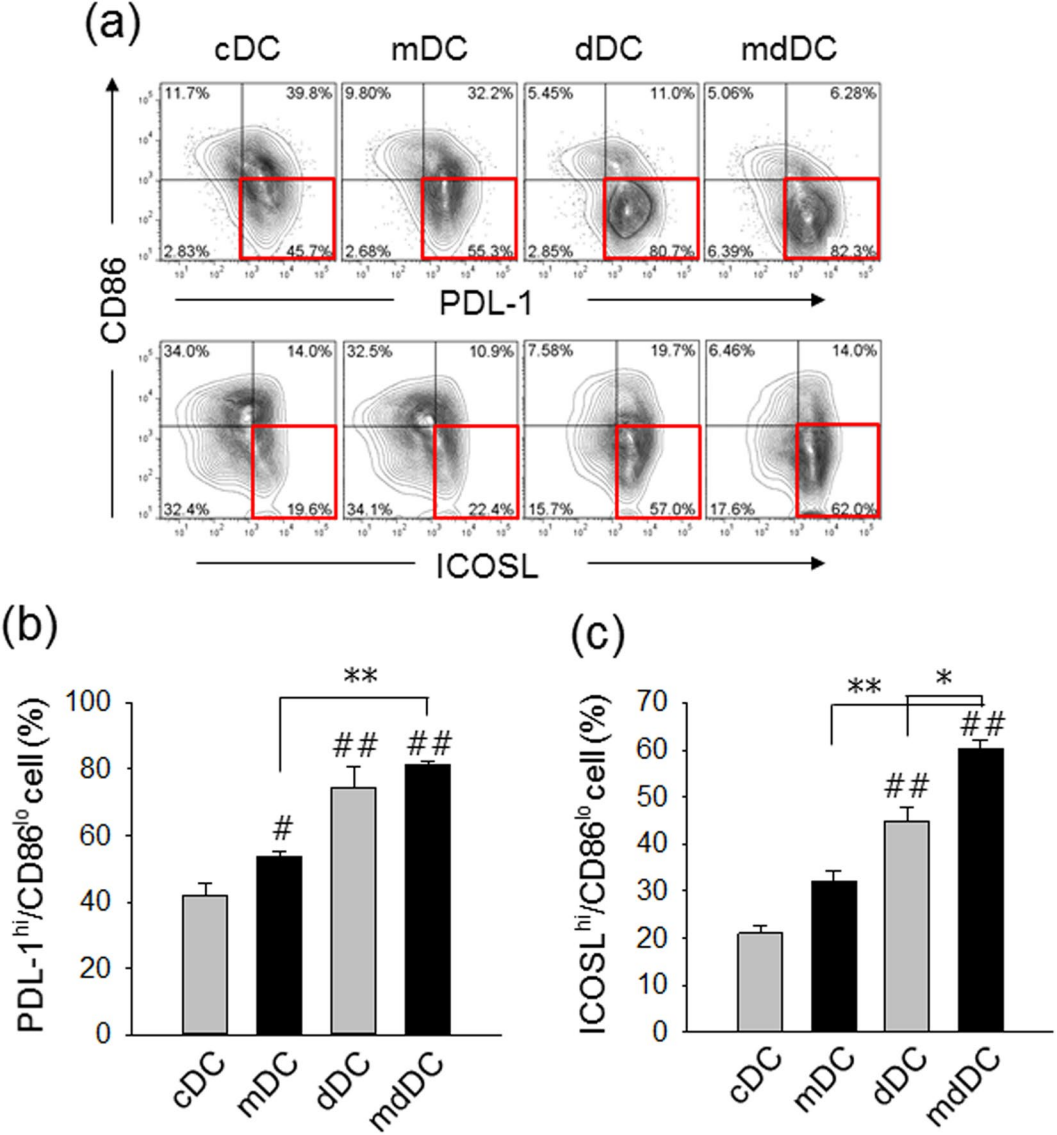
Expression of co-inhibitory molecules by tDCs. (**a**) Mature cDCs, mDCs, dDCs, and mdDCs were analysed for the expression of PDL-1, ICOSL, and CD86 by flow cytometry. Representative histograms are shown. (**b**) The proportion of PDL-1^hi^CD86^low^ cells in each experimental group is shown. (**c**) The proportion of ICOSL^hi^CD86^low^ cells in each experimental group is shown. The data show the mean ± SD of three independent experiments. One way ANOVA tests were performed in order to evaluate significance. ^#^P < 0.05, ^##^P < 0.01 compared with cDC group. *P < 0.05, **P < 0.01 compared with matched group.

We also examined the levels of surface expression of co-inhibitory molecules such as PD-L1 and ICOSL on DCs. The proportion of PD-L1^hi^CD86^low^ cells was significantly higher in mDC (55.2%, P < 0.05), dDC (80.1%, P < 0.01), and mdDC co-cultures (82.8, P < 0.01) than in cDC co-cultures (45.67%) (Fig. 5a,b). The proportion of ICOSL^hi^CD86^low^ cells was also significantly higher in dDC (57.4%, P < 0.01) and mdDC co-cultures (62.6, P < 0.01) than in cDC co-cultures (19.2%) (Fig. 5a,c).

### Pretreatment with MOG35–55 peptide-pulsed minocycline/dexamethasone-conditioned tDCs ameliorates the clinical signs of experimental autoimmune encephalitis (EAE)

**Figure 6:**
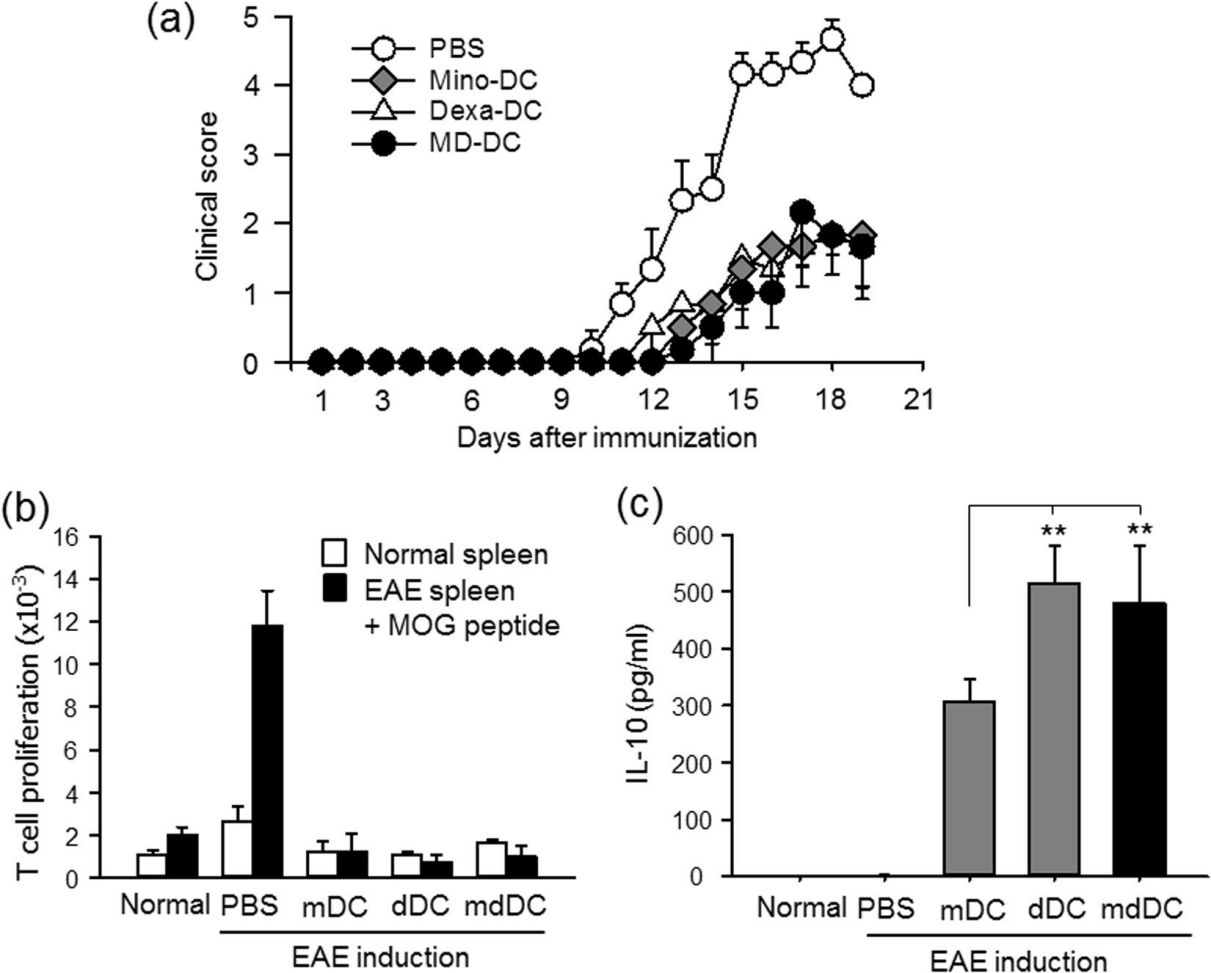
Treatment of EAE using tDCs. (**a**) Mice (n = 6 per group) were subjected to i.v. injection of PBS, or MOG peptide-pulsed mature cDCs, mDCs, dDCs, or mdDCs on day −7 and −3, and EAE was induced on day 0. The clinical score was checked every day. Three independent experiments were performed, and a representative result is shown; the data are expressed as the mean ± SD for at least five animals, i.e., dead mouse was excluded in the statistical analysis. (**b**) Splenocytes were isolated from each group of mice and cultured in the presence or absence of MOG peptides (25 μg/mL) for 3 days. T cell proliferation was induced by incorporating [^3^H]-thymidine before the final 18 h of culture. (**c**) IL-10 secretion in the experimental setting of (**b**) was determined by ELISA. The data show the mean ± SD of three independent experiments. One way ANOVA tests were performed in order to evaluate significance. **P < 0.01 compared with the mDC group.

A murine EAE model was used to examine the regulatory functions of DCs *in vivo*. Female C57BL/6 mice were injected with MOG35–55 peptide-pulsed DCs on days −7 and −3, and EAE was induced on day 0. Control mice received MOG peptide-pulsed cDCs or PBS on the same days. Pretreatment with MOG peptide-pulsed mDCs, dDCs, and mdDCs significantly reduced the clinical signs of EAE (Fig. 6a). There was no discernible difference between the EAE-preventing capabilities of mDCs, dDCs, and mdDCs. To compare the levels of MOG peptide-specific T cells in the mice, splenocytes were isolated from each group of mice and cultured in the presence or absence of MOG peptides (25 μg/mL) for 3 days. Significant MOG peptide-specific T cell proliferation was observed only in the splenocyte cultures of PBS-treated mice (Fig. 6b). The culture supernatants from the splenocyte cultures were assessed for the presence of IL-10 as well. The results showed that splenocytes of mDC-injected mice produced significantly higher amounts of IL-10 than those of PBS-injected mice (Fig. 6c). Splenocytes of dDC-injected mice produced even higher amounts of IL-10 than those of mDC-injected mice. We found no difference in the IL-10-producing capabilities of the splenocytes of dDC-injected and mdDC-injected mice.

## Discussion

Ag-pulsed tDCs are considered promising tools for cellular therapies against autoimmune diseases and graft rejection, as they induce Ag-specific tolerance^12,13^. The therapeutic efficacy of *ex vivo*-generated tDCs has been shown in animal models of autoimmune diseases such as collagen-induced arthritis^20–22^, diabetes^23,24^, and EAE^25^, as well as in animal models of graft rejection^26,27^. One of the major challenges of tDC-based immunotherapy is the optimisation of the protocol to obtain the maximum number of functionally stable tDCs. The combined use of minocycline and dexamethasone to generate *ex vivo* tDCs appeared to be optimal for this process. Minocycline, when used with GM-CSF and IL-4, is known to increase the generation of DCs with tolerogenic properties from mouse bone marrow cells^19^. Dexamethasone is also known to induce the generation of tDCs with potent tolerogenicity from mouse bone marrow cells and from human monocytes when used with GM-CSF and IL-4^17,18^. tDCs generated with dexamethasone express low levels of co-stimulatory molecules and MHC class II molecules, produce elevated levels of IL-10 and lower levels of IL-12, and induce the generation of Tregs^11,17,18,28–32^. Dexamethasone also induces the generation of tolerogenic macrophages^18^. Moreover, the tDCs generated with dexamethasone retain their tolerogenicity for several days, up to a week, even after the dexamethasone is removed^17,18^. A major drawback in generating tDCs with dexamethasone is the reduction in cell recovery, especially when it is added at the initiation of the culture^17^. Combining minocycline, a second-generation semi-synthetic tetracycline with over 30 years of clinical use as an antibiotic, with dexamethasone is an ideal solution to this problem. The addition of minocycline to the dexamethasone-treated culture increased the generation of DCs to about 220% (P < 0.01), compared to dexamethasone-only culture. It is to be noted that in our experiments, minocycline was added from the initiation of the culture, while dexamethasone was added from day 3. The effect of dexamethasone is dependent upon the dose and time of exposure; high concentrations of dexamethasone increase the tolerogenicity, but decrease the cell recovery^17,18^. The cell recovery in dexamethasone-conditioned cultures was significantly and profoundly increased by adding minocycline, and was not reduced even though a high concentration of dexamethasone (1 μM vs. 0.1 μM) was used. This was the reason why we used 1 μM rather than 0.1 M dexamethasone to generated mdDCs.

The tolerogenicity of mdDCs were better than or at least equal to that of the tDCs generated with either one of these agents. mdDCs were more deficient in inducing antigen-specific T cell proliferation, expression of co-stimulatory molecules (CD80 and CD86), and proinflammatory cytokine (TNF-α, IL-1β, IL-12) production than mDCs, or dDCs. However, mdDCs expressed molecules involved in immune suppression, such as IL-10, PD-L1, and ICOSL, more than mDCs, or dDCs. The Treg-inducing activity of mdDCs was also higher than that of mDCs, or dDCs. In contrast to the superiority of mdDCs in *in-vitro* tolerogenic properties, their ability to prevent EAE development was similar to that of mDCs and dDCs. Collectively, the combination of minocycline with dexamethasone generated tDCs with potent tolerogenicity. Another advantage of using this combination is that both the drugs are inexpensive and have been proven to be safe in the clinic.

The mechanism underlying the growth-promoting effect of minocycline on DCs is still unknown. Minocycline has been shown to be beneficial in the treatment of various diseases, including autoimmune disorders such as rheumatoid arthritis, inflammatory bowel disease, scleroderma, aortic aneurysms, and periodontitis, although the mechanism of action is not yet fully understood^33–36^. The efficiency of minocycline against the above-mentioned diseases appears to be mediated by its anti-inflammatory and immunosuppressive effects. Minocycline inhibits the production of proinflammatory cytokines such as TNF-α, IL-1β, and IL-6^37–39^ and matrix metalloproteinases^40^, the processing and presentation of Ags^41^, the expression of MHC class-II molecules^42^, and the proliferation and activation of T cells^43–45^. Our finding that minocycline promoted the growth of DCs was unexpected^19^. As shown in this study, minocycline also exerted growth-promoting effects on DCs conditioned with relatively toxic doses of rapamycin, vitamin D3, or IL-10.

In conclusion, the present study showed that a combination of minocycline and dexamethasone, along with GM-CSF and IL-4, was optimal for maximising the number of tDCs generated *ex vivo*. The tDCs generated with minocycline and dexamethasone exhibited tolerogenic properties that were better than or at least equal to those generated with either one of these agents. We are currently analysing the molecular targets of minocycline on DCs.

## Methods

### Mice

Female C57BL/6 and BALB/c mice (8–12 weeks old) were purchased from Orient Co. Ltd. (Seoul, Korea). All animal care and experimental procedures were approved by the Animal Care Committee of Chungbuk National University, and all experiments were performed in accordance with relevant guidelines and regulations.

### Generation of tDCs from bone marrow cells

DC_S_ were generated as described previously^19^. Briefly, the bone marrow cells obtained from mouse femurs were cultured in 6-well plates (5 × 10^6^ cells/well) in a culture medium with 40 ng/ml GM-CSF and 40 ng/ml IL-4 (both from Creagene, Seongnam, Korea). To generate mDCs, 5 μM minocycline (Santa Cruz Biotechnology, Santa Cruz, CA, USA) was added at the start of the culture. After 3 days, the non-adherent cells were removed by gently shaking the dish and then replacing the medium. To generate the other populations of tDCs, the indicated amounts of rapamycin (Sigma–Aldrich, St. Louis, MO, USA), vitamin D3 (Sigma–Aldrich), IL-10 (PeproTech, Rocky Hill, NJ, USA), and dexamethasone (Sigma-Aldrich) alone and in combination with minocycline, were added on day 3 after removing the non-adherent cells. On day 4, the non-adherent cells were again removed by the same method. On day 6, half the culture medium was replaced with fresh medium. The DCs were harvested by gentle pipetting on day 7. To induce maturation or cytokine production, DCs were exposed to 50 ng/mL IFN-γ and 50 ng/mL TNF-α (both from PeproTech) for 24 h, except in the experiments that induce TNF-α production from DCs. For TNF-α production, DCs were stimulated with LPS (100 ng/ml) for 24 h.

### Preparation of OVA-microspheres

Microspheres containing OVA (OVA-microspheres) were prepared using a homogenisation/solvent evaporation method that has been described previously^19^. Briefly, 400 μl of 50 mg/ml OVA in water was mixed with 2 ml of 100 mg/ml poly(lactic-co-glycolic acid) in ethyl acetate. The OVA content was determined using the Micro Bicinchoninic Acid assay kit (Pierce, Rockford, IL, USA) after lysing the microspheres in a lysis buffer (0.1% SDS and 0.1 N NaOH). Fluorescence-labelled microspheres were prepared by adding fluorescein isothiocyanate (FITC, 5 μg/ml) to the ethyl acetate phase.

### MHC class II-restricted Ag presentation assay

The MHC class II-restricted exogenous Ag presentation assay was performed, as described previously^19^, using OVA-specific CD4^+^ T cell hybridoma DOBW cells, which recognise OVA[323–339]-I-A^d^ complexes and secrete IL-2 in response.

### Phagocytic activity

FITC-labelled OVA-microspheres (1 mg/well OVA) were added to the DCs; after 2 h, the non-phagocytosed microspheres were removed by washing twice with pre-warmed PBS. The cells were harvested, fixed with 1% paraformaldehyde in PBS, and analysed using flow cytometry.

### Cytokine production

The amounts of TNF-α, IL-1β, IL-6, IL-12 p40, and IL-10 in the culture supernatants were measured using commercial ELISA kits (BD Biosciences, San Jose, CA, USA). The amount of TGF-β was measured using an ELISA kit of R&D systems (Abingdon, UK) after treating the culture supernatant with Sample Activation Kit 1 (R&D systems) as suggested by the supplier.

### Mixed lymphocyte reaction

To assay the lymphocyte proliferation in the mixed lymphocyte reaction, allogeneic CD4^+^ T cells seeded in 96-well round bottomed plates (2 × 10^5^ cells/well) were stimulated with DCs (1 × 10^5^ cells/well) for 72 h. DNA synthesis was measured by incorporating [^3^H]-thymidine, which was added before the final 18 h of culture.

### *In vitro* generation of CD4^+^ Tregs from naïve CD4^+^ CD25^−^ T cells

Naïve CD4^+^CD25^−^ T cells were isolated from the spleens of OT-II mice using a CD4^+^ CD25^−^ T cell isolation kit (Miltenyi Botec Inc.). Both tDCs and cDCs were generated from BM cells of C57BL/6 mice, matured in the presence of 50 ng/mL IFN-γ and 50 ng/mL TNF-α for 24 h, and then pulsed with the OVA323–339 peptide (10 μg/ml) for 1 h. After washing with PBS, the DCs (2 × 10^4^ cells/well) were co-cultured with purified OT-II CD4^+^ CD25^−^ T cells (2 × 10^5^ cells/well) for 4 days in a medium containing 100 U/ml of recombinant human IL-2 (PeproTech Inc.). In allogeneic MLR, the DCs (2 × 10^4^ cells/well) generated from BM cells of C57BL/6 mice were cultured with naïve CD4^+^CD25^−^ T cells (2 × 10^5^ cells/well) isolated from the spleens of BALB/c mice for 4 days in a medium containing 10 U/ml of recombinant human IL-2 (PeproTech Inc.). In IL-10 blocking experiments, anti-IL-10 monoclonal antibody, or isotype-matched monoclonal antibody (10 μg/ml, both from BD Biosciences, San Jose, CA, USA) was added from the initiation of the culture.

### Flow cytometry analysis

Cells were stained, as described previously^19^, with monoclonal antibodies against mouse cell surface markers, I-A^b^, CD80, CD86, CD11c, CD4, CD25, and Foxp3, and an isotype-matched control antibody (BD Biosciences). For intracellular Foxp3 staining, cells were permeabilised using the BD Cytofix/Cytoperm Plus kit, according to the manufacturer’s instructions. Subsequent analyses were performed using the FlowJo software (TreeStar, Ashland, OR, USA).

### Induction of EAE and pretreatment with MOG35–55 peptide-pulsed DCs

The DCs were pulsed with 25 μg/mL MOG35–55 peptide for 1 h at 37 °C, harvested by gentle pipetting, washed, and injected into the female C57BL/6 mice (1.2 × 10^6^ cells/mouse, i.v.) on days −7 and −3; EAE was then induced on day 0. On the day of EAE induction, the mice were immunised s.c. with 100 μg of the MOG35–55 peptide in 100 μl of PBS, emulsified in 100 μl of complete Freund’s adjuvant containing 4 mg/mL *Mycobacterium tuberculosis* (H37Ra, Difco/BD Bioscience). In addition, 200 ng of pertussis toxin was injected i.p. on days 0 and 2. EAE paralysis in mice was scored as follows: 0, no symptoms; 1, flaccid tail; 2, hind limb weakness; 3, partial hind limb paralysis; 4, complete hind limb paralysis; 5, moribund state^46^.

### Statistical analysis

An ANOVA test was performed in order to check for significance as stated in the figure legend. Significance was considered when p < 0.05 or lower.

## Electronic supplementary material

Supplementary Information
