# Supplementary material for: Tolerogenic dendritic cells are efficiently generated using minocycline and dexamethasone
Source: Sci Rep. 2017 Nov 8;7:15087. doi: 10.1038/s41598-017-15569-1 (PMC5678112; doi:10.1038/s41598-017-15569-1)
Supplement: Supplementary file 1 — Supplementary Information [file 41598_2017_15569_MOESM1_ESM.pdf]

Tolerogenic dendritic cells are efficiently generated using minocycline and dexamethasone

Jae-Hee Lee[1,+], Chan-Su Park[1,+], Sundong Jang[1,+], Ji-Wan Kim[1], Sang-Hyeon Kim[1], Sukgil Song[1], Kyungjae Kim[2], and Chong-Kil Lee[1,*]

[1]College of Pharmacy, Chungbuk National University, Cheongju 28644, South Korea

[2]College of Pharmacy, Sahmyook University, Seoul 01795, South Korea

[*] Corresponding author. cklee@chungbuk.ac.kr

[+] These authors contributed equally in this work

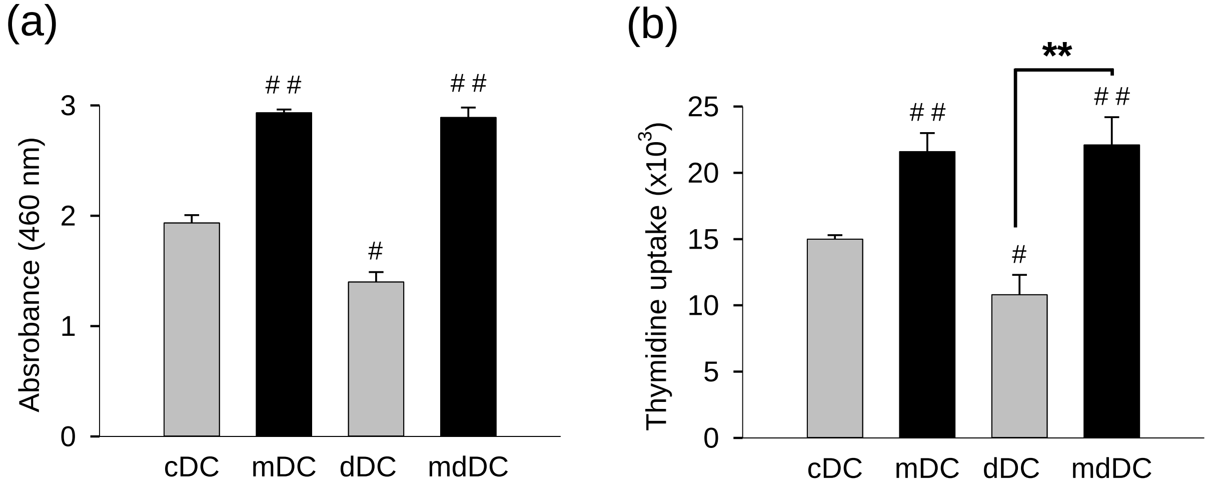

Supplementary Figure S1. Growth-promoting activity of minocycline. (**a**) Immature cDCs, mDCs, dDCs, or mdDCs generated from BM cells of C57BL/6 mice were harvested on day 4 from the initiation of culture, washed, and added wells of 96-well plate (2× 10^5 cells/well) in a culture containing minocycline (5 μM, for mDCs), dexamethasone (1 μM, for dDCs), or both (for mdDCs) together with 40 ng/ml GM-CSF and 40 ng/ml IL-4. After culturing for 24 h, 50 μl of a solution containing 1 mg/ml XTT (2,3-bis(2-methoxy-4-nitro-5-sulfophenyl)-2H-tetrazolium-5-carboxanilide) (Sigma-Aldrich) and 0.383 mg/ml phenazine methosulfate (PMS, Fluka, Buchs, Switzerland) diluted in PBS were added to each well and incubated for 4 h at 37 ℃.. Absorbance was measured at 460 nm. (**b**) Immature cDCs, mDCs, dDCs, or mdDCs generated from BM cells of C57BL/6 mice were harvested on day 4 from the initiation of culture, washed, and added wells of 96-well plate (2× 10^5 cells/well) in a culture containing minocycline (5 μM, for mDCs), dexamethasone (1 μM, for dDCs), or both (for mdDCs) together with 40 ng/ml GM-CSF and 40 ng/ml IL-4. After incubating for 18 h, cells were pulsed with 1 μCi/ml [^{3}H]thymidine (Du Pont, Boston, MA, USA) for an additional 6 h. After harvesting the cells, [^{3}H]thymidine uptake was measured using a scintillation counter. The data are presented as the mean ± SD of three independent experiments performed in triplicate cultures. One way ANOVA tests were performed in order to evaluate significance. $^{\#}$ $P < 0.05$, $^{\#\#}$ $P < 0.01$ compared with cDC group. **$P < 0.01$ compared with matched group.

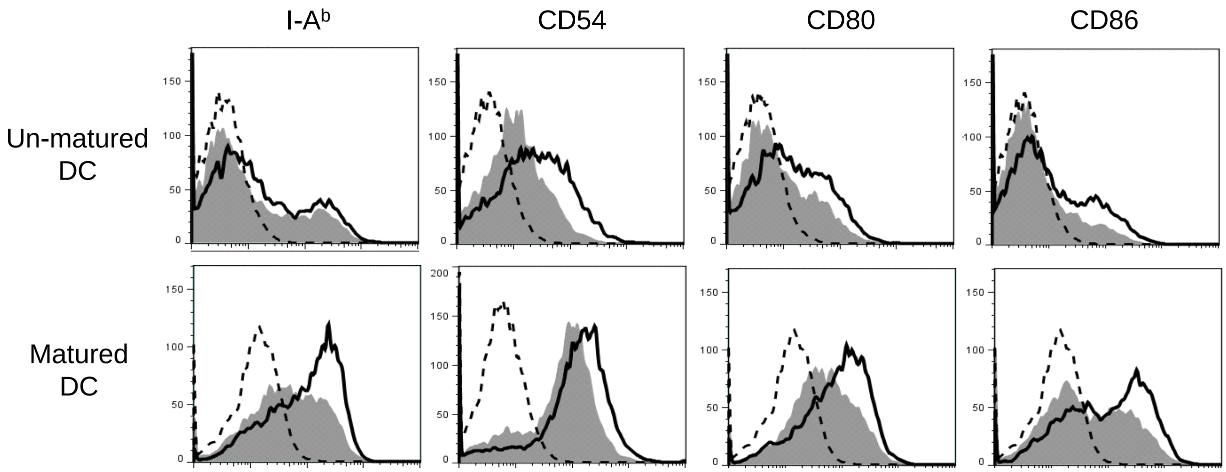

Supplementary Figure S2. mdDCs are refractory to maturation. cDCs and mdDCs were harvested on day 6 from the initiation of the culture (un-matured DC), or exposed to 50 ng/mL IFN-γ plus 50 ng/mL TNF-α for 24 h to induce maturation (matured DC), and analyzed by flow cytometry for the expression of I-A^b, CD54, CD80 and CD86. cDCs, black line; mdDCs, shaded area; isotype-matched control, dotted line. A representative set of histograms from 2 separate experiments was shown.
